# Temporal partitioning and the potential for avoidance behaviour within South African carnivore communities

**DOI:** 10.1002/ece3.10380

**Published:** 2023-08-15

**Authors:** Kyle Smith, Jan A. Venter, Mike Peel, Mark Keith, Michael J. Somers

**Affiliations:** ^1^ Mammal Research Institute, Department of Zoology and Entomology University of Pretoria Pretoria South Africa; ^2^ Department of Conservation Management, Faculty of Science, George Campus Nelson Mandela University George South Africa; ^3^ ARC‐Animal Production Institute Rangeland Ecology Group Nelspruit South Africa; ^4^ School for Animal, Plant and Environmental Sciences University of the Witwatersrand Johannesburg South Africa; ^5^ Applied Behavioural Ecology and Ecosystem Research Unit University of South Africa Florida South Africa

**Keywords:** activity, camera traps, Carnivora, overlap, South Africa, temporal avoidance

## Abstract

Carnivora occupy many ecological niches fundamental to ecosystem functioning. Within this diverse order, carnivore species compete to establish dominance, ensure survival and maintain fitness. Subordinate carnivores must, therefore, adapt their behaviour to coexist with dominant species. One such strategy is the partitioning of temporal activity patterns. We aim to determine interspecific avoidance patterns among sympatric carnivores by examining coexistence along a temporal axis. We compared the temporal activity patterns of 13 carnivore species using multi‐seasonal camera trapping data from four protected areas across South Africa: Associated Private Nature Reserves, Madikwe Game Reserve, Mountain Zebra National Park and Tswalu Kalahari Reserve. Interspecific coefficients of overlap in diel and core activity periods were calculated over the study period and during the wet and dry seasons. Furthermore, interspecific spatiotemporal behaviour was examined using time‐to‐event analyses. Our results showed that complete avoidance of diel activity patterns was rare among South African carnivore species. Most species were predominantly nocturnal and, therefore, diel activity overlap was high, whereas core activity overlap was significantly lower (*p* < .001). Diel activity overlap was significantly lower during the dry than wet seasons (*p* = .045). Lastly, evidence of spatiotemporal aggregation revolved around scavenging species. We show the importance of seasonality in the temporal avoidance behaviours of South African carnivores while highlighting the need for fine‐scaled behavioural analyses. Overall, we show that the daily activity patterns of most subordinate South African carnivore species are not influenced by top‐down forces in the form of competitional suppression and risk exerted by dominant species. If avoidance is required, it is more likely to manifest as fine‐scaled avoidance of core activity periods. We suggest that the focus on core activity periods might be a more suitable tool for interspecific temporal partitioning research.

## Graphical abstract

Using camera trapping data from four protected areas across South Africa, we determined the avoidance patterns among sympatric carnivore species by examining coexistence along a temporal axis. Our results showed that carnivore species can coexist without drastic changes to their diel activity patterns and, if temporal avoidance is required at the population level, it is more likely to manifest as fine‐scaled avoidance of core activity periods. We also show the importance of seasonality in altering interspecific avoidance behaviours of carnivore species.

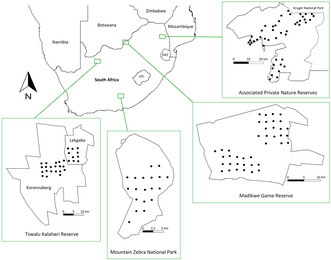

## 1 INTRODUCTION

Carnivores (species of the order Carnivora) are integral to an ecosystem's trophic structure and are, therefore, vital to ecosystem functioning (Elmhagen et al., 2010; Estes et al., 2011; Hoeks et al., 2020). They occupy various levels within this trophic hierarchy, and many species fill the role of both predator and prey (Palomares & Caro, 1999). Sympatric carnivores, therefore, likely experience different degrees of competition and risk, from indirect exploitation of limited resources to interference competition that involves direct antagonistic interactions, such as kleptoparasitism, depredation and territorial killings (Caro & Stoner, 2003; Linnell & Strand, 2000; Palomares & Caro, 1999). Outcomes of interspecific competition among carnivores are usually dictated by body size, predatory behaviour, morphological adaptations, age structure, social organisation and species diversity within the region (Donadio & Buskirk, 2006; Lehmann et al., 2017; Linnell & Strand, 2000). Even though interspecific avoidance behaviour is adaptive and manifested over evolutionary timescales, subordinate carnivores must often adjust their behaviour to ensure coexistence with more dominant carnivores due to population‐wide changes of, for instance, demographics and sex ratios (Kronfeld‐Schor & Dayan, 2003; Lehmann et al., 2017; Lima & Dill, 1990; Linnell & Strand, 2000; Trinkel & Kastberger, 2005).

Avoidance behaviour constitutes many dimensional considerations related to niche theory, ranging from spatial, temporal and diet partitioning, to reliance on reactive or predictive decision making in response to various forms of interspecific competition and risk (Broekhuis et al., 2013; Carothers & Jaksic, 1984; Hayward & Kerley, 2008; Linnell & Strand, 2000). Spatial partitioning may force subordinate species to occupy areas with unfavourable resource availability (Ritchie & Johnson, 2009). In response, these species may resist exclusion from favourable habitat by being active at different times of the day, reducing the likelihood of encounters with the dominant species (Swanson et al., 2014).

Temporal avoidance behaviour is a form of resource partitioning whereby animal species, constrained by morphological characteristics and adaptations, are active at different periods of the 24‐h day to reduce the risk posed by species that occupy higher levels of the dominance hierarchy (Schoener, 1974; see review by Bennie et al., 2014; Hayward & Slotow, 2009; Kronfeld‐Schor & Dayan, 2003). Temporal partitioning is, thus, a behavioural adaptation that species could employ to coexist with other species deemed a threat to their survival or fitness (Carothers & Jaksic, 1984).

In this study, we used data from Snapshot Safari's extensive camera‐trapping surveys within four South African protected areas (Pardo et al., 2021) to assess temporal partitioning as a strategy for interspecific avoidance behaviour among carnivore species. Examining multiple carnivore communities from environmentally diverse regions will allow for a better understanding of the many different species' temporal behaviours.

Due to their opportunistic predatory behaviour, felids (e.g. lions *Panthera leo*, leopards *Panthera pardus* and caracals *Caracal caracal*) are responsible for most intraguild killings (Curveira‐Santos et al., 2022; Donadio & Buskirk, 2006). We, thus, hypothesise that subordinate carnivore species will more likely avoid felids, which could potentially be a greater risk for their survival than the other species. Leopards have been observed killing carnivore species as small as genets (Curveira‐Santos et al., 2022). From this, we predict that subordinate carnivore species of all body sizes will show signs of avoiding leopards. Interspecific antagonism and killing are not only limited to large carnivores but are also observed among mesopredators. For example, caracals have been shown to kill smaller carnivore species such as African wildcats *Felis silvestris lybica* and mongooses (Curveira‐Santos et al., 2022). Avoidance behaviour is, therefore, predicted to also manifest among the smaller carnivore species.

Pronounced seasonal variations in environmental characteristics are experienced within South Africa due to its higher latitudes (Daan & Aschoff, 1975). These differences range from unimodal rainfall seasons that result in clear seasonal differences in vegetation quality (i.e. wet and dry seasons), to changes in the availability of resources that could affect the behaviour of carnivores. For example, diet overlap between bat‐eared foxes *Otocyon megalotis* and aardwolves *Proteles cristata* is likely to occur only during the colder months of the year, when aardwolves shift a considerable portion of their diet to *Hodotermes* termites due to less availability of the preferred *Trinervitermes* termites (Kamler et al., 2013; Williams et al., 1997). As a result, the two species may compete during winter. In addition, Kamler et al. (2017) showed that bat‐eared foxes have significantly larger group sizes during the dry seasons in the Northern Cape, South Africa. This was proposed as a possible response of bat‐eared foxes to increased temporal overlap with black‐backed jackals *Lupulella mesomelas* in the reserve during the dry season (Kamler et al., 2017). Furthermore, Périquet et al. (2021) showed that seasonality plays an important role in the facilitative and competitional relationship between lions and spotted hyaenas *Crocuta crocuta*, as resource availability varies between the wet and dry seasons, and spotted hyaenas are more likely to actively hunt prey during the dry season, making them less dependent on scavenging lion kills. Ultimately, we predict that indications of avoidance behaviours among many carnivore species will differ between the wet and dry seasons.

Lastly, most of Southern Africa's carnivore species are nocturnal (Comley et al., 2020; de Satgé et al., 2017; Greco et al., 2021; Vissia & van Langevelde, 2022; Webster et al., 2021). Carnivore species that conform behaviourally to a similar temporal niche classification at the population level (e.g. nocturnal, crepuscular) will have high temporal overlap values when diel activity is compared. However, temporal avoidance may still be used to coexist, which necessitates finer‐scaled temporal comparisons of activity. Therefore, we predict that interspecific temporal avoidance behaviour among the carnivores in this study will more likely manifest as finer‐scaled asynchronization of their core activity periods than complete avoidance throughout the 24‐h diel period.

## 2 MATERIALS AND METHODS

### 2.1 Study areas

**FIGURE 1:**
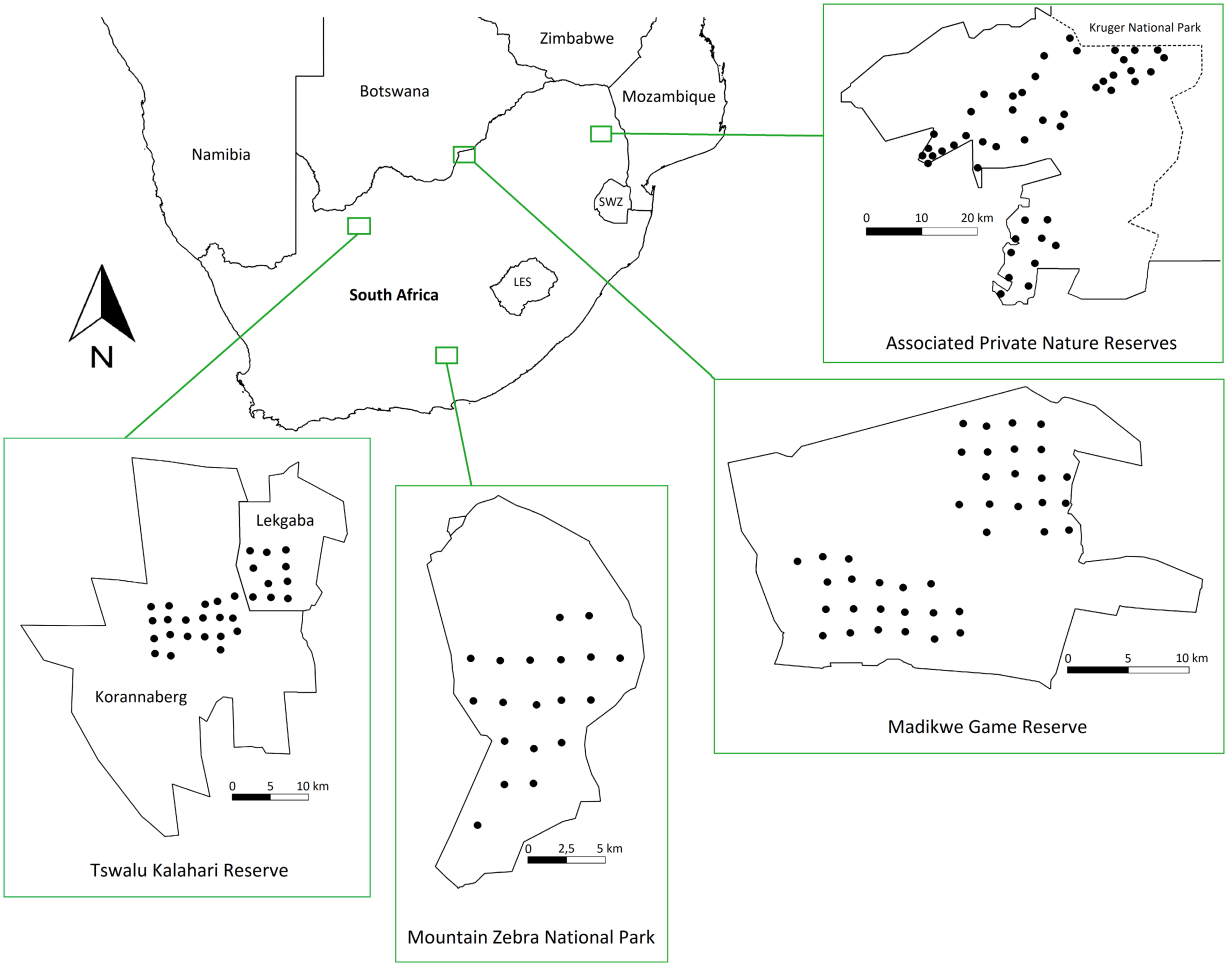
Snapshot Safari's camera trapping networks in the four South African protected areas. Each protected area's fenced borders are represented by solid lines, whereas unfenced borders are represented by stippled lines. The dots in each protected area indicate the locations of camera traps.

Snapshot Safari's camera trapping data from four protected areas across South Africa were included in this study (Pardo et al., 2021; Figure 1; Table 1): Associated Private Nature Reserves (APNR), Madikwe Game Reserve (MGR), Mountain Zebra National Park (MZNP) and Tswalu Kalahari Reserve (TKR). These protected areas span a considerable latitudinal and longitudinal range, allowing for variation in climatic conditions, environmental characteristics and ecological diversity. Apart from APNR where camera traps were placed according to vegetation differences, the protected areas followed a standardised camera trapping system where cameras were placed facing game paths at the centre of a grid network of 5 km^2^ per camera placement (see Pardo et al., 2021 for a more detailed description of the camera‐trap network, Snapshot Safari). Camera trapping deployments ran for multiple seasons and years (Table 1).

**TABLE 1:** Characteristics and deployment data of the Snapshot Safari camera trap networks in four South African protected areas: Associated Private Nature Reserves (APNR), Madikwe Game Reserve (MGR), Mountain Zebra National Park (MZNP) and Tswalu Kalahari Reserve (TKR).

	APNR	MGR	MZNP	TKR
Size (km^2^)	2055	750	214	1100
Fencing	Fenced border, but open to Kruger National Park along eastern border	Fenced border	Fenced border	Fenced border, separated into two fenced camps^a^
Biome	Semi‐arid savanna	Semi‐arid savanna	Albany thicket, Nama‐Karoo and grassland	Arid Kalahari savanna
Rainfall seasons	Summer	Summer	Summer	Summer
Wet seasons	Oct–Apr	Oct–Apr	Oct–May	Oct–Apr
Dry seasons	May–Sep	May–Sep	Jun–Sep	May–Sep
Annual rainfall	Approx. 500 mm	Approx. 500 mm	Approx. 400 mm	Approx. 360 mm
Number of cameras	49	40	19	30^b^
Camera deployment	Jun 2017–Oct 2019	Aug 2017–Oct 2019	Aug 2017–Oct 2019	Nov 2018–Nov 2019
Camera days^c^	33,000	15,900	7900	9800

^a^ Korannaberg, the main section of TKR, and Lekgaba, which supports TKR's lion population.

^b^ 20 cameras in Korannaberg and 10 in Lekgaba.

^c^ The number of cumulative days (24‐h cycles) that all cameras were active.

### 2.2 Analysis

We only included Carnivora species with at least 20 independent detections within a specific protected area in the analyses. Photo‐captures were rendered independent by limiting the time interval between subsequent photo‐captures of the same species at a specific camera station to a minimum of 60 min, reducing the possibility of pseudoreplication (Niedballa et al., 2019).

#### 2.2.1 Interspecific activity overlap

We compared the daily activity patterns of species populations derived from kernel density estimates within the specific protected areas. This was done by calculating the coefficient of overlap ($\hat{∆}$) and the associated 95% smoothed bootstrapped confidence intervals with 10,000 resamples using the *overlap* package (Meredith & Ridout, 2021; Ridout & Linkie, 2009) in R (v4.0.3; R Core Team, 2021). The coefficient of overlap is a proportional value representing the possible similarity in species' diel activity patterns and ranges from 0 to 1, indicating completely different and identical activity patterns, respectively. According to Meredith and Ridout (2021), when at least one species in a pair obtained <50 photo‐captures, the overlap estimator ${\hat{∆}}_{1}$ was calculated, and when both species obtained more than 50 photo‐captures, ${\hat{∆}}_{4}$ was calculated.

We also calculated the core activity periods (50% core isopleths) and their overlap between species using the *circular* package (Agostinelli & Lund, 2022) in R and the highest species‐specific bandwidth estimation within each species‐pair. An appropriate bandwidth value for each species was calculated with a maximum moment, kmax = 3.

To determine whether there are differences between diel and core activity overlaps, we compared the collective diel activity overlap and core activity overlap values for all the carnivore species‐pairs using paired Wilcoxon tests (*α* = 0.05) with the *wilcox.test* function in R. Using the same test, we determined the seasonal differences in overlap values by comparing the wet and dry season's collective diel activity overlaps, as well as the two seasons' collective core activity overlaps.

The two separated sections of TKR, Lekgaba (lions present) and Korannaberg (lions absent) present a unique opportunity where the behaviour of a mammal species can be compared within the same region with similar environmental characteristics, but where lions are present and absent. Therefore, to test the effect of lion presence on the daily activity patterns of carnivore species in TKR, we used similar analyses as described above to compare each carnivore species' diel activity pattern separately between the Lekgaba (lions present) and Korannaberg (lions absent) sections of TKR.

#### 2.2.2 Fine‐scaled spatiotemporal behaviour between species

We performed a time‐to‐event analysis, derived from Karanth et al. (2017) and Watabe et al. (2022), to determine spatiotemporal avoidance or aggregation between carnivore species. In this context, the spatiotemporal dimension refers to differences in temporal use within a shared space. The analysis entailed extracting the time period from each photo‐capture of a specific species (reference species) to the nearest single photo‐capture, before or after, of the comparing species (proximate species) at shared camera trapping stations. A maximum of 7 days before and after each reference detection was used to include proximate detections, as this allowed for enough comparisons among species while still likely maintaining biological relevance. To increase reliability, we only included species‐pairs with at least 20 total comparisons across all camera stations within 7 days before and after the reference detections. We then calculated the observed median of all time intervals between reference and proximate detections within the 7‐day relevance period.

We then applied a randomisation process to each photo‐capture of the proximate species. This entailed randomly selecting a camera station, date and time from the original dataset for the specific proximate species, thereby preserving biological relevance in activity period preferences. This was repeated 1000 times to generate 1000 randomised datasets against which the reference species detections were compared in the time‐to‐event analysis. The median of the minimum time between proximate and reference detections for each 1000 randomised datasets was then calculated and plotted as a density distribution of medians. Similar to standard permutation tests, we calculated a proportional value (*p*‐value) as p=n/N, where n is the number of randomised medians greater than the observed median and N is the total number of randomised medians for the specific species‐pair. A two‐tailed significance level (*α* = 0.05) was considered, where a *p* > .975 indicated a significant possibility of spatiotemporal aggregation and *p* < .025 indicated a significant possibility of avoidance behaviour.

## 3 RESULTS

Within each of the sites, six species obtained 20 or more independent photo‐captures (Table 2). Therefore, we were able to look at a total of 13 species across the four protected areas, with approximately half being mesocarnivores. The body sizes of these species range from the largest predator, lions, to the small mesopredator, African wildcats.

**TABLE 2:** The number of independent Snapshot Safari camera trap photo‐captures (>60 min apart) for South African Carnivora species within the Associated Private Nature Reserves (APNR), Madikwe Game Reserve (MGR), Mountain Zebra National Park (MZNP) and Tswalu Kalahari Reserve (TKR).

Site	Carnivora species	Total	Wet	Dry
APNR	Spotted hyaena *Crocuta crocuta*	1712	853	859
	Lion *Panthera leo*	190	97	93
	Leopard *Panthera pardus*	165	82	83
	African civet *Civettictis civetta*	72	20	52
	African wild dog *Lycaon pictus*	46	17	29
	Honey badger *Mellivora capensis*	24	15	9
	Genet *Genetta* spp.	19	7	12
	White‐tailed mongoose *Ichneumia albicauda*	16	10	6
	Black‐backed jackal *Lupulella mesomelas*	15	3	12
	Side‐striped jackal *Lupulella adustus*	13	8	5
	Caracal *Caracal caracal*	7	3	5
	African wildcat *Felis silvestris lybica*	3	0	3
	Dwarf mongoose *Helogale parvula*	2	2	0
	Banded mongoose *Mungos mungo*	1	0	1
	Cheetah *Acinonyx jubatus*	1	0	1
	Meller's mongoose *Rhynchogale melleri*	1	0	1
MGR	Brown hyaena *Hyaena brunnea*	216	86	130
	Spotted hyaena *Crocuta crocuta*	173	78	95
	Black‐backed jackal *Lupulella mesomelas*	70	24	46
	Leopard *Panthera pardus*	44	17	27
	Lion *Panthera leo*	36	12	24
	African wildcat *Felis silvestris lybica*	25	7	18
	Banded mongoose *Mungos mungo*	13	11	2
	Genet *Genetta* spp.	10	4	6
	Slender mongoose *Galerella sanguinea*	10	2	8
	Caracal *Caracal caracal*	8	4	4
	African civet *Civettictis civetta*	8	3	5
	Honey badger *Mellivora capensis*	7	4	3
	African wild dog *Lycaon pictus*	6	4	2
	Aardwolf *Proteles cristata*	4	2	2
	Cheetah *Acinonyx jubatus*	4	2	2
	Serval *Leptailurus serval*	4	1	3
	White‐tailed mongoose *Ichneumia albicauda*	2	2	0
MZNP	Black‐backed jackal *Lupulella mesomelas*	816	456	368
	Aardwolf *Proteles cristata*	175	119	59
	Brown hyaena *Hyaena brunnea*	108	76	32
	Bat‐eared fox *Otocyon megalotis*	57	23	34
	Lion *Panthera leo*	49	19	30
	Caracal *Caracal caracal*	23	14	9
	Small‐spotted genet *Genetta genetta*	19	14	5
	Cheetah *Acinonyx jubatus*	6	4	2
	Cape grey mongoose *Herpestes pulverulentus*	6	4	2
	Meerkat *Suricata suricatta*	5	5	0
	Striped polecat *Ictonyx striatus*	4	4	0
	Yellow mongoose *Cynictis penicillate*	3	1	2
	Water mongoose *Atilax paludinosus*	2	2	0
	Black‐footed cat *Felis nigripes*	1	0	1
	African striped weasel *Poecilogale albinucha*	1	0	1
TKR	Bat‐eared fox *Otocyon megalotis*	260	149	111
	Black‐backed jackal *Lupulella mesomelas*	234	134	100
	Brown hyaena *Hyaena brunnea*	85	45	40
	Cape fox *Vulpes chama*	44	20	24
	African wildcat *Felis silvestris lybica*	36	19	17
	Aardwolf *Proteles cristata*	21	12	9
	Cheetah *Acinonyx jubatus*	16	9	7
	Meerkat *Suricata suricatta*	13	9	4
	African wild dog *Lycaon pictus*	12	5	7
	Caracal *Caracal caracal*	12	9	3
	Lion *Panthera leo*	9	5	4
	Leopard *Panthera pardus*	6	5	1
	Yellow mongoose *Cynictis penicillata*	4	3	1
	Genet *Genetta* spp.	3	2	1
	Striped polecat *Ictonyx striatus*	3	1	2

*Note*: The shaded species were included in the analyses.

### 3.1 Interspecific overlap in temporal activity patterns

**FIGURE 2:**
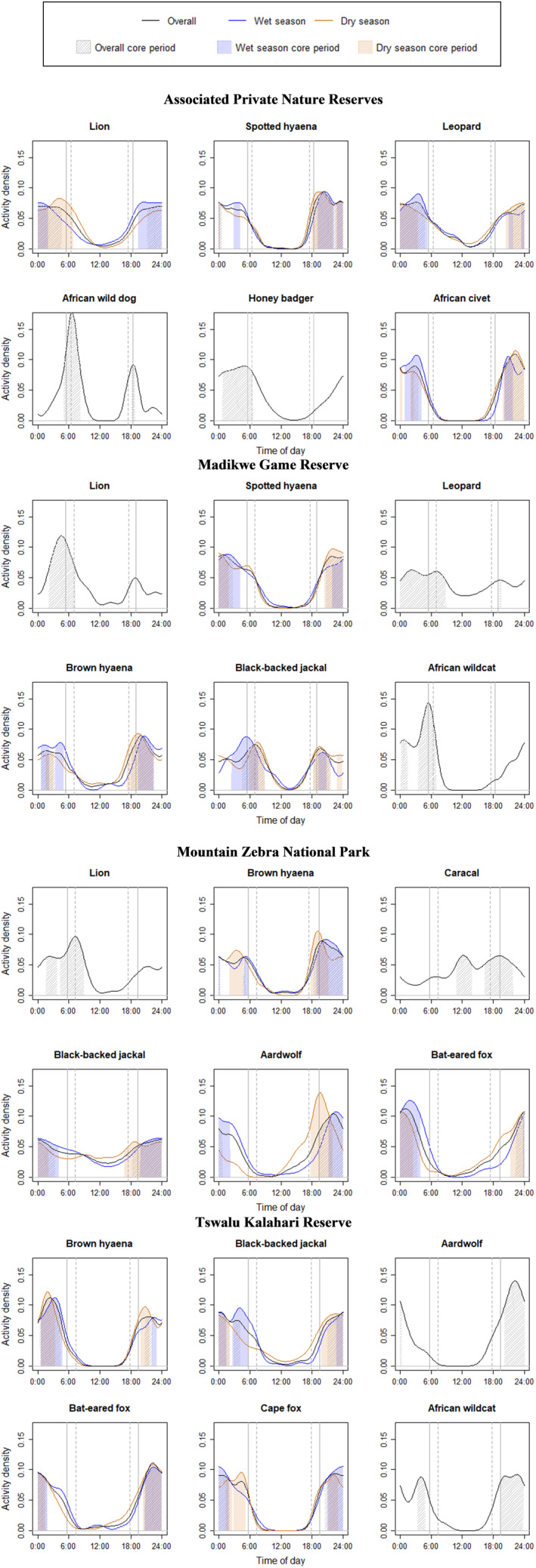
Diel activity patterns of carnivores from the four South African protected areas. Sunrise and sunset during the wet seasons are represented by solid vertical lines, and during the dry seasons by stippled vertical lines. Times of sunrise and sunset within each protected area were sourced from the NOAA (National Oceanic and Atmospheric Administration) Global Monitoring Laboratory's NOAA Solar Calculator.

The majority of the species assessed within the four protected areas were primarily nocturnal (Figure 2). Exceptions included APNR's African wild dogs *Lycaon pictus*, and MGR's lions and black‐backed jackals that were most active during crepuscular hours, and MGR's leopards and MZNP's caracals that were active throughout the 24‐h period (i.e. cathemeral).

**FIGURE 3:**
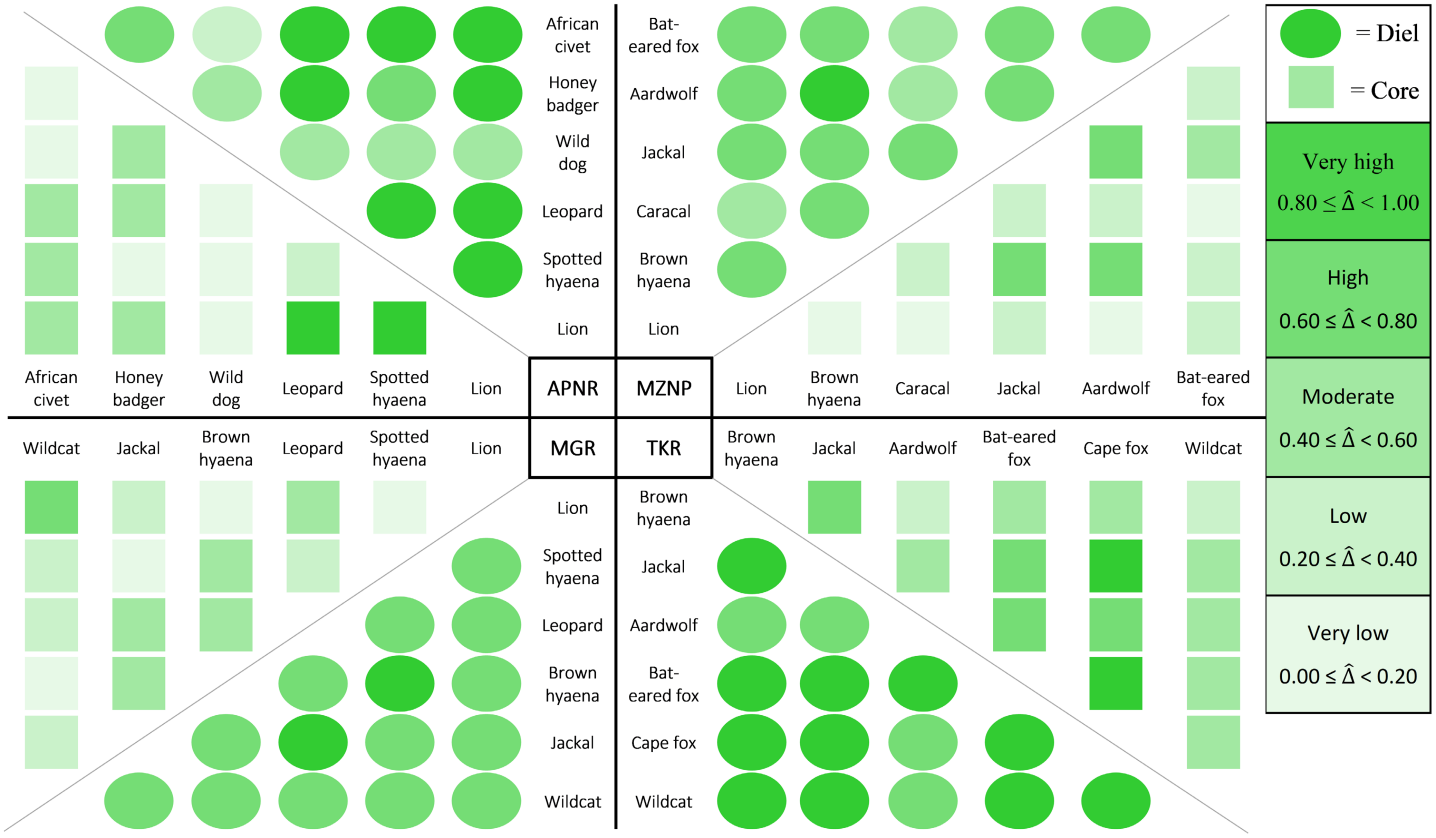
The temporal coefficient of overlap ($\hat{∆}$) in activity patterns of carnivore species from South Africa's Associated Private Nature Reserves (APNR), Madikwe Game Reserve (MGR), Mountain Zebra National Park (MZNP) and Tswalu Kalahari Reserve (TKR).

**FIGURE 4:**
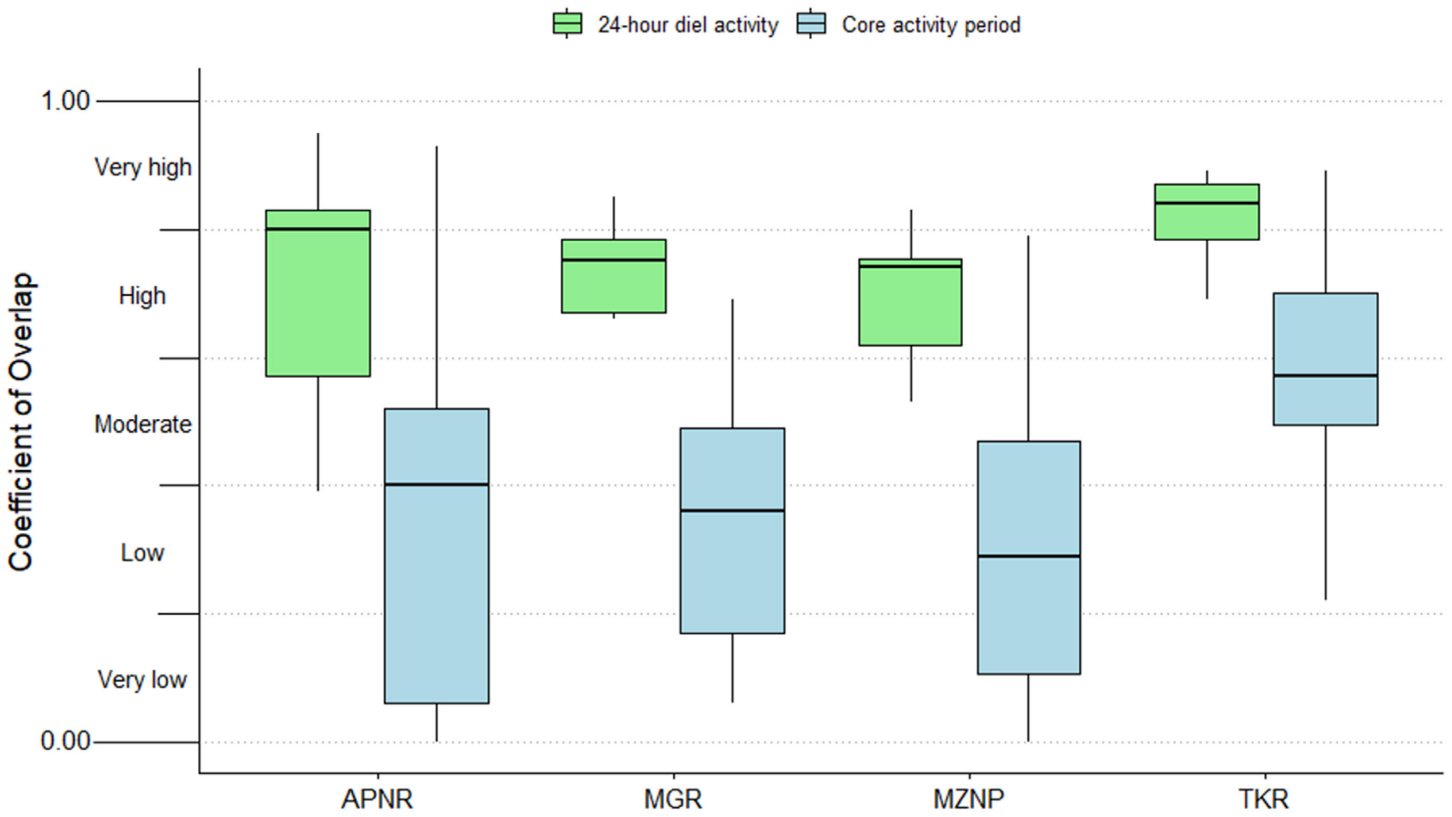
The comparison between 24‐h diel and core activity overlaps of carnivore species within South Africa's Associated Private Nature Reserves (APNR), Madikwe Game Reserve (MGR), Mountain Zebra National Park (MZNP) and Tswalu Kalahari Reserve (TKR).

The majority (87%) of the 60 species‐pairs had high (0.60 ≤ ${\hat{∆}}_{\text{diel}}$ < 0.80, 48.3%) to very high (0.80 ≤ ${\hat{∆}}_{\text{diel}}$ < 1.00, 38.3%) coefficients of overlap in their diel activity patterns throughout the entire study period within the respective protected areas (Figures 3 and 4; Table A1). In contrast, most (80%) of the 60 species‐pairs had moderate (0.40 ≤ ${\hat{∆}}_{\text{core}}$ < 0.60, 31.7%), low (0.20 ≤ ${\hat{∆}}_{\text{core}}$ < 0.40, 25.0%) and very low (0.00 ≤ ${\hat{∆}}_{\text{core}}$ < 0.20, 23.3%) coefficients of overlap in core activity periods throughout the entire study period (Figures 3 and 4; Table A1). Overall, the overlap of the carnivore species' core activity periods (${\hat{∆}}_{\text{core}}$) was significantly lower (*V* = 1711, *p* < .001) than the overlap of their 24‐h diel activity patterns (${\hat{∆}}_{\text{diel}}$).

More specifically, APNR's African wild dogs showed moderate to low temporal overlap with most other carnivore species. This was especially noticeable when core activity periods were compared (Figure 3; Table A1). The APNR's other large predators showed very high temporal overlap, except for the low ${\hat{∆}}_{\text{core}}$ observed between leopards and spotted hyaenas. Furthermore, despite APNR's honey badgers *Mellivora capensis* having high ${\hat{∆}}_{\text{diel}}$ with spotted hyaenas and African civets *Civettictis civetta*, the two pairs had very low ${\hat{∆}}_{\text{core}}$.

All of the carnivore species assessed within MGR had high to very high ${\hat{∆}}_{\text{diel}}$ (Figures 3 and 4; Table A1). However, MGR's two hyaena species had very low ${\hat{∆}}_{\text{core}}$ with lions, whereas the reserve's leopards also had low ${\hat{∆}}_{\text{core}}$ with lions. Despite having a high ${\hat{∆}}_{\text{diel}}$ with the reserve's two apex predators, lions and spotted hyaenas, MGR's black‐backed jackals had a low ${\hat{∆}}_{\text{core}}$ with lions and a very low ${\hat{∆}}_{\text{core}}$ with spotted hyaenas. African wildcats, the smallest carnivore species assessed in MGR, had low ${\hat{∆}}_{\text{core}}$ with all the other species except lions.

Most (12 of 15) of the carnivore species‐pairs assessed in MZNP had high to very high ${\hat{∆}}_{\text{diel}}$ (Figures 3 and 4; Table A1). Caracals paired with lions, aardwolves and bat‐eared foxes were exceptions, with only moderate overlap. Furthermore, despite having high ${\hat{∆}}_{\text{diel}}$, MZNP's brown hyaenas *Hyaena brunnea* and lions had no overlap in their core activity periods throughout the entire study period. In contrast, MZNP's black‐backed jackals, brown hyaenas and aardwolves had high ${\hat{∆}}_{\text{core}}$, while bat‐eared foxes and black‐backed jackals had moderate ${\hat{∆}}_{\text{core}}$.

Most (11 of 15) of the carnivore species‐pairs assessed in TKR had very high ${\hat{∆}}_{\text{diel}}$, with aardwolves paired with brown hyaenas, black‐backed jackals, Cape foxes *Vulpes chama* and African wildcats having high ${\hat{∆}}_{\text{diel}}$ (Figures 3 and 4; Table A1). In addition, the majority (13 of 15) of carnivore species‐pairs assessed in TKR had a moderate or higher ${\hat{∆}}_{\text{core}}$; only brown hyaenas paired with aardwolves and African wildcats had a low ${\hat{∆}}_{\text{core}}$. Lastly, TKR's Cape foxes had very high ${\hat{∆}}_{\text{diel}}$ and ${\hat{∆}}_{\text{core}}$ with the reserve's bat‐eared foxes and black‐backed jackals.

Only brown hyaenas (55 detections in Korannaberg and 30 in Lekgaba), black‐backed jackals (151 detections in Korannaberg and 83 in Lekgaba) and bat‐eared foxes (240 detections in Korannaberg and 20 in Lekgaba) recorded enough detections to have their species‐specific temporal activity patterns compared between the two sections. Species‐specific overlap in diel activity patterns for brown hyaenas (${\hat{∆}}_{\text{diel}}$ = 0.85, 95% CI = 0.71–0.96), black‐backed jackals (${\hat{∆}}_{\text{diel}}$ = 0.94, 95% CI = 0.86–1.00) and bat‐eared foxes (${\hat{∆}}_{\text{diel}}$ = 0.86, 95% CI = 0.71–0.96) were very high when each of the species' diel activity patterns was separately compared between the Lekgaba (lions present) and the Korannaberg sections (lions absent) of TKR. In addition, each of these species' core activity periods was highly similar in the two sections of the reserve (brown hyaenas' ${\hat{∆}}_{\text{core}}$ = 0.61, black‐backed jackals' ${\hat{∆}}_{\text{core}}$ = 0.79, bat‐eared foxes' ${\hat{∆}}_{\text{core}}$ = 0.78).

### 3.2 Seasonality in interspecific temporal overlap

**FIGURE 5:**
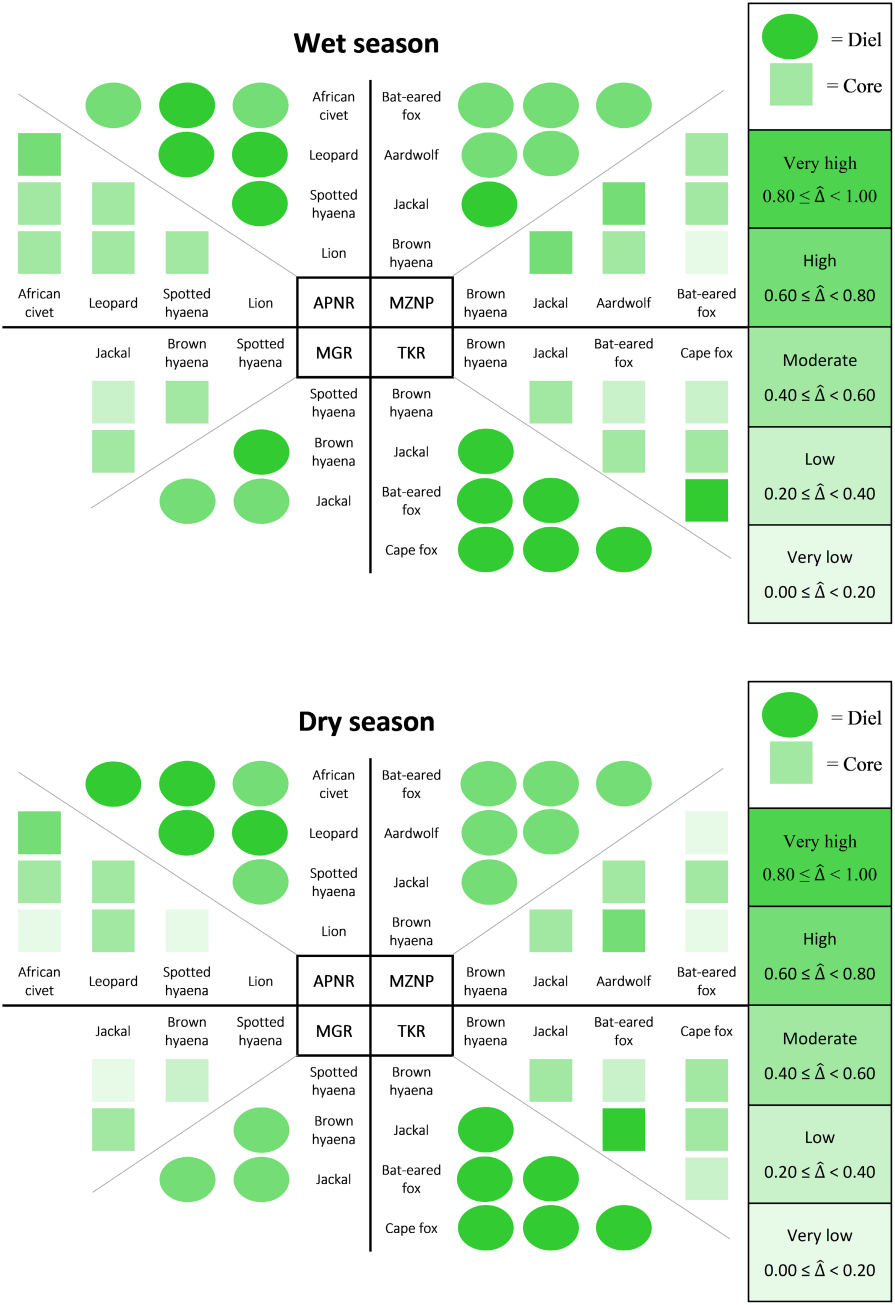
The temporal coefficient of overlap ($\hat{∆}$) in activity patterns of carnivore species during the wet and dry seasons within South Africa's Associated Private Nature Reserves (APNR), Madikwe Game Reserve (MGR), Mountain Zebra National Park (MZNP) and Tswalu Kalahari Reserve (TKR).

**FIGURE 6:**
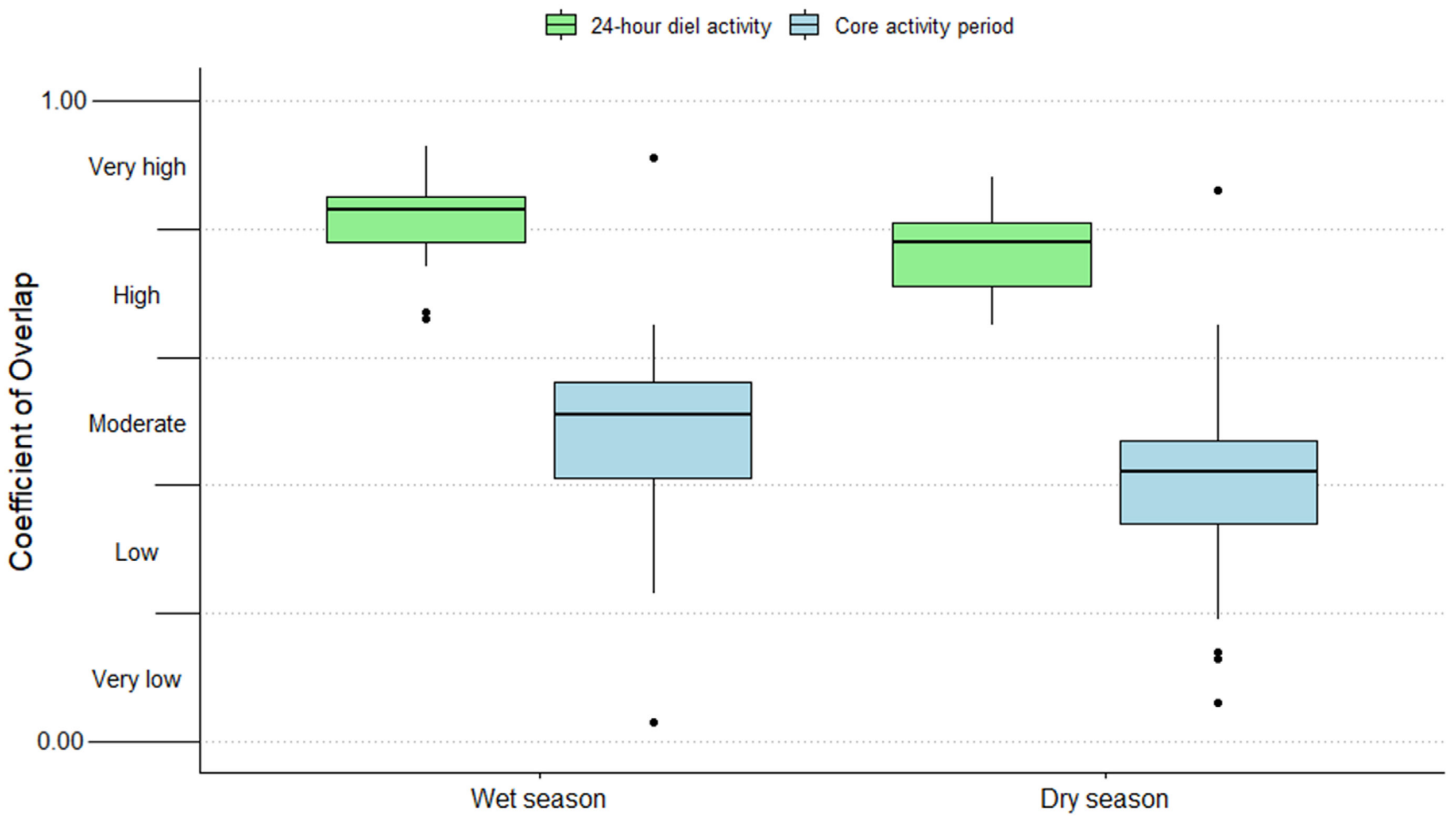
Seasonal comparisons of total diel and core activity overlap of carnivore species from South Africa's Associated Private Nature Reserves, Madikwe Game Reserve, Mountain Zebra National Park and Tswalu Kalahari Reserve.

Most of the species‐pairs assessed within the protected areas had very high ${\hat{∆}}_{\text{diel}}$ during the wet seasons and high ${\hat{∆}}_{\text{diel}}$ during the dry seasons (Figures 5 and 6; Table A2). Although marginal, this resulted in a significant difference in ${\hat{∆}}_{\text{diel}}$ between the two seasons (*V* = 173.5, *p* = .045). However, with the test statistic again being marginal, there was no significant difference in ${\hat{∆}}_{\text{core}}$ between the two seasons (*V* = 170.5, *p* = .058).

All of the carnivore species assessed within APNR had high to very high ${\hat{∆}}_{\text{diel}}$ during both seasons (Figure 5; Table A2). Furthermore, these species all had moderate ${\hat{∆}}_{\text{core}}$ during the wet seasons, except for African civets paired with leopards which had a high ${\hat{∆}}_{\text{core}}$. During the dry seasons, the ${\hat{∆}}_{\text{core}}$ of APNR's lions paired with spotted hyaenas, and African civets decreased to a very low level, whereas APNR's African civets maintained a high ${\hat{∆}}_{\text{core}}$ with the reserves' leopards.

Most species assessed within MGR had high ${\hat{∆}}_{\text{diel}}$ during the wet and dry seasons, with only spotted hyaenas paired with brown hyaenas having a very high ${\hat{∆}}_{\text{diel}}$ (Figure 5; Table A2). Furthermore, all of MGR's species assessed during both seasons experienced a decline in ${\hat{∆}}_{\text{core}}$ during the dry season. Most notably, the ${\hat{∆}}_{\text{core}}$ of brown hyaenas and spotted hyaenas decreased from a moderate ${\hat{∆}}_{\text{core}}$ during the wet season to a low ${\hat{∆}}_{\text{core}}$ during the dry season. MGR's black‐backed jackals had high ${\hat{∆}}_{\text{diel}}$ with spotted hyaenas during both seasons, but low and very low ${\hat{∆}}_{\text{core}}$ during the wet and dry seasons, respectively.

All of the species that were assessed during MZNP's wet and dry seasons had high or very high ${\hat{∆}}_{\text{diel}}$ (Figure 5; Table A2). Most of these species also had moderate to high ${\hat{∆}}_{\text{core}}$. However, bat‐eared foxes paired with brown hyaenas in MZNP had very low ${\hat{∆}}_{\text{core}}$ during both seasons, as did bat‐eared foxes paired with aardwolves, but only during the dry seasons (Figure 5; Table A2). The most noticeable seasonal difference in ${\hat{∆}}_{\text{core}}$ within MZNP was between aardwolves and bat‐eared foxes, with 87% less overlap during the dry compared to wet seasons.

All of TKR's species that were assessed during the wet and dry seasons had very high ${\hat{∆}}_{\text{diel}}$ (Figure 5; Table A2). Most of these species also had moderate or higher ${\hat{∆}}_{\text{core}}$, with low ${\hat{∆}}_{\text{core}}$ observed between TKR's brown hyaenas and bat‐eared foxes during both seasons, brown hyaenas and Cape foxes during the wet season, and between the two fox species during the dry season. Cape foxes and bat‐eared foxes had very similar activity patterns during the wet seasons, with a marked decrease during the dry seasons, especially in ${\hat{∆}}_{\text{core}}$. In contrast, bat‐eared foxes and black‐backed jackals had substantially more overlap during the dry than wet seasons (Figure 5; Table A2).

### 3.3 Spatiotemporal behaviour

**FIGURE 7:**
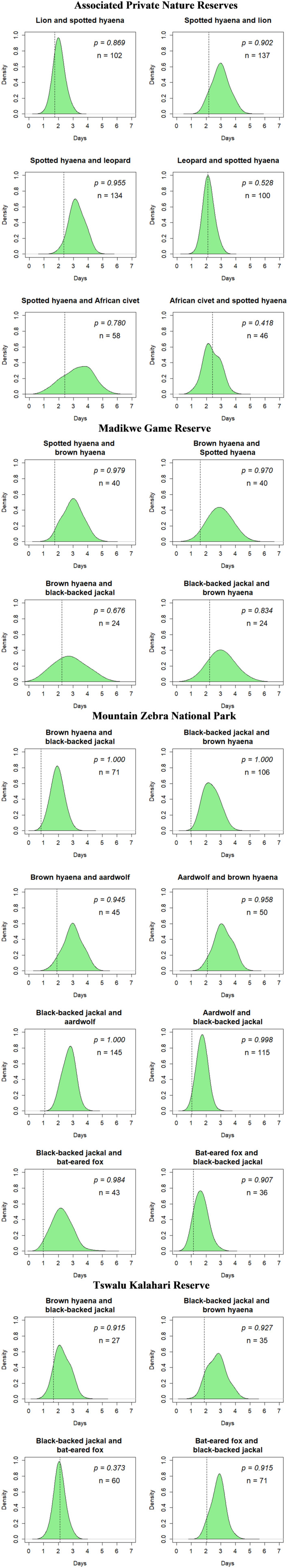
Relative spatiotemporal behaviour of carnivore species within the four South African protected areas. The first‐mentioned species are the reference species within the pair. The shaded density distribution represents 1000 randomised medians of time between reference and proximate detections, whereas the vertical stippled line represents the median observed time between reference and proximate detections. The *p*‐value shows the proportional number of randomised medians greater than the observed median and *n* represents the total number of proximate detections obtained within the 7‐day period before and after the reference detections.

Only 11 species‐pairs across all four protected areas obtained enough (≥20) proximate photo‐captures within 7 days of the reference detections. None of the species‐pairs showed significant spatiotemporal segregation (*p* < .025), whereas only four species‐pairs displayed significant spatiotemporal aggregation (*p* > .975; Figure 7). These include MGR's spotted hyaenas and brown hyaenas, and MZNP's black‐backed jackals paired with brown hyaenas, aardwolves and bat‐eared foxes. Despite most of the species‐pairs not obtaining significant *p*‐values that would show either aggregation or segregation, the majority had *p*‐values closer to indications of aggregation (*p* > .5) than segregation (*p* < .5; Figure 7).

## 4 DISCUSSION

In this study, we compared the activity patterns of South African Carnivora species to investigate temporal partitioning as a potential avoidance mechanism to reduce encounters with more dominant carnivores. However, most species‐pairs (63%) across all combinations and sites in this study showed no clear indications of using temporal avoidance behaviour as a strategy to coexist with a potential risk‐associated, dominant species. A trade‐off exists in subordinate carnivores between resource acquisition and risk associated with sympatric predators (Linnell & Strand, 2000), with the former likely outweighing the latter in most cases in this study. This is a viewpoint shared by many previous studies on African carnivores, in which hunting success is said to be prioritised over the possibility of encountering dominant predators (see Balme, Pitman, et al., 2017; Cozzi et al., 2012; Miller et al., 2018; Mugerwa et al., 2017; Müller et al., 2022).

Nocturnality in many large carnivores is attributed to increased concealment and thermal stress avoidance (Miller et al., 2018; Rabaiotti & Woodroffe, 2019), which generally leads to increased hunting success (Mugerwa et al., 2017; Van Orsdol, 1984). Furthermore, Greco et al. (2021) rejected the hypothesis that carnivores' temporal activity patterns are dictated by apex predator avoidance and instead attributed it to optimal niche utilisation and prey acquisition (i.e. hunting and foraging behaviour). A lack of temporal avoidance behaviour has also been reported for mesopredators and small carnivores (Mills et al., 2019; Vissia & van Langevelde, 2022). Negative influences exerted on subordinate carnivores by larger, more dominant carnivores are said to be an exception rather than a commonly observed rule (Comley et al., 2020). The findings of this study partially support the statement made by Comley et al. (2020), as clear patterns of temporal partitioning with dominant species were rarely observed among subordinate carnivores. For example, our findings show that bat‐eared foxes do not temporally avoid black‐backed jackals and that regular encounters between the two species seem very likely, particularly during the dry seasons. Results from this study, therefore, support Kamler et al. (2017), who proposed that significantly larger group sizes of bat‐eared foxes during dry seasons is a possible response to increased temporal overlap with black‐backed jackals. In addition, our results support Kamler et al. (2013) in showing that the activity patterns of bat‐eared foxes are not influenced by a common risk‐associated species such as black‐backed jackals. Furthermore, black‐backed jackals pose a significant threat to Cape foxes and will kill them in territorial defence, leading to the suppression of Cape fox populations (Kamler et al., 2013). Conversely, TKR's Cape foxes showed no evidence of temporally avoiding black‐backed jackals, which contradicts previous findings (Edwards et al., 2015; Kamler et al., 2012). Bat‐eared foxes and Cape foxes have little dietary overlap and do not recognise each other as a noticeable antagonistic threat (Kamler et al., 2012). Therefore, avoidance behaviour between the two fox species is highly unlikely and was not observed in this study as both fox species had very similar daily activity patterns. In addition, our findings support the idea that leopards use strategies other than temporal partitioning to coexist with other large carnivores (Miller et al., 2018). One such method is the characteristic caching behaviour of leopards, in which they hoist prey carcasses into trees to avoid kleptoparasitic losses, lending support to the kleptoparasitism‐avoidance hypothesis (Balme, Miller, et al., 2017; MacDonald, 1976). Furthermore, this study supports Ramesh et al. (2017), who showed that smaller carnivores rarely use temporal partitioning to avoid large carnivores.

Notably, the presence of lions within the Lekgaba section of TKR did not affect the activity patterns of brown hyaenas, black‐backed jackals and bat‐eared foxes. We suggest two possible explanations: (1) Mesocarnivore suppression in terms of temporal behaviour is comparable among lions and other carnivore species that fill the role of apex predators when lions are removed from an ecosystem (e.g. Korannaberg's African wild dogs), or (2) the apex predators do not have an impact on the diel activity patterns of smaller subordinate carnivore species. Ultimately, the removal of lions in a region where other large carnivore species, such as African wild dogs and cheetahs *Acinonyx jubatus* remain, are unlikely to change the temporal behaviour of subordinate species. This is applicable to species that may rely on scavenging opportunities (i.e. TKR's brown hyaenas and black‐backed jackals) and smaller species that are at risk of intraguild killings (i.e. TKR's bat‐eared foxes). We encourage further research into the matter.

A lack of clear temporal avoidance behaviour does not imply a lack of competition or risk, but encounters with dominant species may be low enough to be considered negligible (Romero‐Muñoz et al., 2010). It is acknowledged that other avoidance methods, such as spatial or dietary partitioning, may instead be used to facilitate coexistence. True encounter frequencies between elusive carnivores are nearly impossible to determine using camera trapping methods and, thus, require finer‐scaled continuous data collection methods such as GPS collaring and direct observations. Knowledge of site‐specific carnivore densities is also important because low densities result in the rarity of encounters, which may make temporal avoidance negligible (Mills, 2015; Müller et al., 2022; Romero‐Muñoz et al., 2010). As the densities of the species increase, so will the number of encounters between them (Creel et al., 2001). This may have been the case in the significant spatiotemporal aggregation between MZNP's aardwolves and black‐backed jackals. Documented cases of aardwolves being attacked and killed by black‐backed jackals are lacking (Curveira‐Santos et al., 2022), and there is a negligible overlap in their diets (Klare et al., 2010). Therefore, it can only be assumed that this aggregation was due to factors other than interspecific attraction, such as the high detection frequency of black‐backed jackals or an attraction to similar habitat with high productivity. However, we recommend that future research should aim to refine the time‐to‐event method of interspecific spatiotemporal analyses by examining the effect of detection frequencies and population densities on outcomes, the effect of multiple species occurring after a reference detection, and the chosen maximum time between reference and proximate detections as longer periods may present issues of randomisation. These are potential limitations to obtaining reliable results from which robust inferences could be made.

The potential for competition between specific carnivore species is also important when determining possible temporal avoidance behaviour (Caro & Stoner, 2003). Even though competition between most sympatric carnivore species is theoretically acknowledged, it should be empirically confirmed through direct observations of antagonistic behaviour. For instance, inter‐species killings have been documented (Palomares & Caro, 1999), but these events may be rare enough to be considered negligible. For example, aardwolves have rarely been observed as victims of aggressive interactions and have no dietary overlap with dominant carnivores (Curveira‐Santos et al., 2022); thus, avoidance may not be necessary. Studies regularly overlook documented cases of intraguild aggression among carnivores and rely on theoretical research and assumptions (Curveira‐Santos et al., 2022). There is a clear need for empirical evidence of risk between carnivore species in the scientific literature. This will increase the relevance and reliability of theoretical findings and inferences regarding interspecific competition among carnivores.

We predicted that subordinate carnivore species would avoid felids such as lions and leopards more than other carnivores due to their opportunistic predatory behaviour, which leads to them being responsible for most intraguild killings (Curveira‐Santos et al., 2022; Donadio & Buskirk, 2006). However, our results do not clearly distinguish the prevalence of avoidance behaviour between felids and other carnivore species such as hyaenas and black‐backed jackals and, therefore, does not provide clear support to this hypothesis. In addition, the study does not support the prediction that subordinate carnivores of all body sizes (e.g. APNR's African civets, and MGR's brown hyaenas, black‐backed jackals and African wildcats) will show signs of avoiding leopards temporally. Lions, however, appear to affect the core activity periods of many subordinate carnivore species and, therefore, this study suggests that subordinate carnivores will be more inclined to avoid the core activity periods of lion prides than solitary leopards.

Some species‐pairs displayed clear potential for temporal avoidance behaviour. This mainly revolved around the crepuscularity of the APNR's African wild dogs, and the cathemeral behaviour of MZNP's caracals. African wild dogs are vulnerable to interference competition from larger carnivores such as spotted hyaenas and, in particular, lions (Creel & Creel, 1996). As a result, African wild dogs may have undergone forced evolutionary adaptations for activity preferences during crepuscular periods (Swanson et al., 2014). It should be noted that non‐overlapping activity patterns do not necessarily indicate avoidance behaviour, but that the potential for avoidance behaviour exists.

Most species‐pairs that displayed possible temporal avoidance behaviour in this study were predominantly nocturnal and, thus, likely rely on finer‐scaled temporal partitioning via adaptations to activity peaks and core activity periods to facilitate coexistence. This was evident in our findings as core activity overlap was significantly lower compared to diel activity overlap in most species‐pairs. Our findings, therefore, satisfy the prediction that interspecific temporal avoidance behaviour among South African carnivores will more likely be expressed as finer‐scaled asynchronization of their core activity periods than complete avoidance throughout the 24‐h diel period. This allows species to remain active when hunting success is greatest, while reducing the risk of encounters with dominant species, which may be less costly to manage than large‐scaled avoidance (Broekhuis et al., 2013). For example, brown hyaenas are scavenging specialists and are facilitated by the presence of larger carnivores, such as lions, as they benefit from eating the remains of their kills (Mills, 2015). However, our findings show that brown hyaenas use fine‐scaled partitioning of core activity periods to avoid direct interactions with lions. This finding is supported by Mills and Mills (1982) and Bashant et al. (2020). We also found evidence of fine‐scaled avoidance behaviour in two facultative scavengers; MGR's black‐backed jackals peaked in activity before and after spotted hyaena activity peaks. This study recommends that future research on temporal avoidance behaviour should focus on finer‐scaled avoidance of, for example, core activity periods.

Seasonal considerations are also important when comparing the daily activity patterns of carnivore species (Vilella et al., 2020). We observed a relatively common trend in which the temporal overlap between species was noticeably lower during the dry seasons compared to the wet seasons. This trend has also been reported by Finnegan et al. (2021) within a carnivore assemblage in the Brazilian Pantanal, and by Vissia and van Langevelde (2022) among carnivores in Botswana's central Tuli region. This decrease in temporal overlap during the dry season could be due to increased competition among species as resources become less available and more concentrated in specific areas (Finnegan et al., 2021; Vissia & van Langevelde, 2022). Seasonality in temporal overlap was evident between aardwolves and bat‐eared foxes, with less overlap during the dry compared to the wet seasons. Aardwolves seem to forage for termites earlier in the night than bat‐eared foxes during MZNP's colder months to lower the risk of losing access to termite colonies. Furthermore, MGR's lions and spotted hyaenas had noticeably different diel activity patterns, suggesting competition and the potential for avoidance behaviour. However, double the number of MGR's lion photo‐captures were recorded during the dry season compared to the wet season. Furthermore, there were strong indications of possible temporal avoidance during APNR's dry seasons when the two species had contrasting activity peaks. As a result, avoidance behaviour caused by increased competition between lions and spotted hyaenas may be much more evident during the dry seasons, which may have resulted in the indications of temporal segregation between spotted hyaenas and lions in MGR. This trend is supported by Périquet et al. (2021), who stated that there is a greater possibility of facilitation between lions and spotted hyaenas during the wet seasons when encounters between the two species are mainly centred around carcasses; however, interference competition dominates interactions during the dry seasons when carcasses are more readily available, and, thus, the need for scavenging lion kills is reduced. Seasonality is frequently overlooked in studies concerning the diel activity patterns and interspecific temporal overlap of carnivores. Therefore, it is advised that future studies should not only include multi‐seasonal data and comparisons, but also attempt to maintain an equal balance of detections from each respective season. Taking into account seasonality at high latitudinal regions when considering the interspecific relationships of carnivores is important and encouraged and should receive more focus in future research studying similar aspects of carnivore community ecology.

Due to the paucity of temporal avoidance behaviour observed in this study, we conclude that the daily activity patterns of most South African Carnivora species are likely not influenced by top‐down forces in the form of competitional suppression and risk exerted by more dominant species. However, they are more likely to respond to risk by avoiding periods when dominant carnivore species are most active, rather than complete avoidance of their diel activity patterns. Complete temporal partitioning as an avoidance strategy among South African carnivores should, therefore, be considered a rarity and is more likely to manifest as finer scaled behavioural adjustments.

## AUTHOR CONTRIBUTIONS

**Kyle Smith:** Conceptualization (equal); data curation (equal); formal analysis (lead); investigation (equal); methodology (lead); project administration (equal); writing – original draft (lead); writing – review and editing (lead). **Jan A. Venter:** Conceptualization (equal); data curation (lead); funding acquisition (lead); project administration (supporting); supervision (supporting); writing – review and editing (supporting). **Mike Peel:** Data curation (lead); funding acquisition (equal); project administration (supporting); writing – review and editing (supporting). **Mark Keith:** Conceptualization (supporting); data curation (equal); funding acquisition (supporting); project administration (supporting); supervision (supporting); writing – review and editing (equal). **Michael J. Somers:** Conceptualization (equal); data curation (equal); funding acquisition (equal); project administration (lead); supervision (lead); writing – review and editing (supporting).

## CONFLICT OF INTEREST STATEMENT

There are no conflicts of interest to declare.

## Data Availability

The camera trap data for this study is available on the University of Pretoria's FigShare data repository https://doi.org/10.25403/UPresearchdata.22716733.v1.

## Appendix

**TABLE A1:** The temporal activity overlaps of carnivore species within South Africa's Associated Private Nature Reserves (APNR), Madikwe Game Reserve (MGR), Mountain Zebra National Park (MZNP) and Tswalu Kalahari Reserve (TKR).

APNR	Lion	Spotted hyaena	Leopard	African wild dog	Honey badger	African civet
Lion	–	0.87	0.93	0.00	0.46	0.53
Spotted hyaena	0.87 (0.82–0.92)	–	0.36	0.10	0.17	0.57
Leopard	0.95 (0.89–0.99)	0.86 (0.80–0.91)	–	0.00	0.51	0.49
African wild dog	0.56 (0.44–0.68)	0.53 (0.42–0.64)	0.53 (0.41–0.66)	–	0.40	0.00
Honey badger	0.80 (0.66–0.93)	0.70 (0.56–0.84)	0.81 (0.66–0.93)	0.58 (0.41–0.74)	–	0.02
African civet	0.80 (0.71–0.88)	0.85 (0.77–0.91)	0.80 (0.71–0.89)	0.39 (0.27–0.52)	0.69 (0.52–0.85)	–

MGR	Lion	Spotted hyaena	Leopard	Brown hyaena	Black‐backed jackal	African wildcat
Lion	–	0.08	0.53	0.13	0.36	0.69
Spotted hyaena	0.66 (0.53–0.78)	–	0.29	0.50	0.10	0.21
Leopard	0.78 (0.63–0.91)	0.71 (0.59–0.83)	–	0.48	0.51	0.39
Brown hyaena	0.66 (0.52–0.79)	0.80 (0.72–0.87)	0.78 (0.65–0.89)	–	0.46	0.06
Black‐backed jackal	0.77 (0.62–0.90)	0.75 (0.64–0.85)	0.85 (0.74–0.95)	0.79 (0.69–0.89)	–	0.31
African wildcat	0.72 (0.57–0.85)	0.79 (0.63–0.93)	0.66 (0.50–0.81)	0.67 (0.52–0.81)	0.67 (0.52–0.81)	–

MZNP	Lion	Brown hyaena	Caracal	Black‐backed jackal	Aardwolf	Bat‐eared fox
Lion	–	0.00	0.00	0.21	0.00	0.23
Brown hyaena	0.74 (0.61–0.86)	–	0.39	0.67	0.79	0.25
Caracal	0.56 (0.38–0.74)	0.62 (0.45–0.78)	–	0.34	0.29	0.00
Black‐backed jackal	0.75 (0.63–0.86)	0.79 (0.73–0.86)	0.76 (0.60–0.89)	–	0.64	0.55
Aardwolf	0.62 (0.49–0.74)	0.82 (0.74–0.89)	0.59 (0.42–0.75)	0.75 (0.69–0.80)	–	0.39
Bat‐eared fox	0.65 (0.51–0.78)	0.74 (0.61–0.85)	0.53 (0.36–0.70)	0.73 (0.63–0.82)	0.83 (0.71–0.93)	–

TKR	Brown hyaena	Black‐backed jackal	Aardwolf	Bat‐eared fox	Cape fox	African wildcat
Brown hyaena	–	0.60	0.22	0.43	0.46	0.39
Black‐backed jackal	0.83 (0.75–0.91)	–	0.53	0.79	0.82	0.54
Aardwolf	0.71 (0.54–0.86)	0.69 (0.54–0.84)	–	0.73	0.67	0.59
Bat‐eared fox	0.84 (0.74–0.92)	0.86 (0.79–0.92)	0.81 (0.64–0.93)	–	0.89	0.56
Cape fox	0.89 (0.78–0.97)	0.88 (0.79–0.96)	0.75 (0.57–0.90)	0.89 (0.79–0.97)	–	0.57
African wildcat	0.85 (0.72–0.95)	0.84 (0.71–0.94)	0.76 (0.58–0.91)	0.82 (0.70–0.92)	0.89 (0.75–0.99)	–

*Note*: Below the diagonal: Coefficient of overlap, ${\hat{∆}}_{\text{diel}}$ (95% confidence interval). Above the diagonal: Coefficient of overlap in core activity periods, ${\hat{∆}}_{\text{core}}$.

**TABLE A2:** The temporal activity overlaps of carnivore species during the wet and dry seasons within South Africa's Associated Private Nature Reserves (APNR), Madikwe Game Reserve (MGR), Mountain Zebra National Park (MZNP) and Tswalu Kalahari Reserve (TKR).

APNR	Lion	Spotted hyaena	Leopard	African civet
Wet seasons				
Lion	–	0.56	0.43	0.40
Spotted hyaena	0.90 (0.84–0.96)	–	0.41	0.57
Leopard	0.83 (0.71–0.93)	0.83 (0.74–0.91)	–	0.64
African civet	0.78 (0.64–0.90)	0.86 (0.72–0.95)	0.78 (0.64–0.90)	–
Dry seasons				
Lion	–	0.13	0.47	0.14
Spotted hyaena	0.76 (0.67–0.85)	–	0.43	0.47
Leopard	0.85 (0.74–0.95)	0.88 (0.80–0.94)	–	0.65
African civet	0.73 (0.61–0.84)	0.82 (0.73–0.90)	0.81 (0.70–0.91)	–

MGR	Spotted hyaena	Brown hyaena	Black‐backed jackal
Wet seasons			
Spotted hyaena	–	0.56	0.23
Brown hyaena	0.87 (0.77–0.96)	–	0.53
Black‐backed jackal	0.75 (0.59–0.89)	0.74 (0.59–0.88)	–
Dry seasons			
Spotted hyaena	–	0.34	0.19
Brown hyaena	0.70 (0.60–0.80)	–	0.40
Black‐backed jackal	0.71 (0.58–0.83)	0.78 (0.65–0.88)	–

MZNP	Brown hyaena	Black‐backed jackal	Aardwolf	Bat‐eared fox
Wet seasons				
Brown hyaena	–	0.60	0.51	0.03
Black‐backed jackal	0.82 (0.73–0.91)	–	0.65	0.51
Aardwolf	0.78 (0.67–0.88)	0.74 (0.67–0.82)	–	0.46
Bat‐eared fox	0.67 (0.50–0.82)	0.66 (0.52–0.80)	0.79 (0.63–0.93)	–
Dry seasons				
Brown hyaena	–	0.40	0.61	0.13
Black‐backed jackal	0.72 (0.61–0.82)	–	0.47	0.48
Aardwolf	0.65 (0.48–0.79)	0.65 (0.56–0.75)	–	0.06
Bat‐eared fox	0.72 (0.55–0.86)	0.71 (0.59–0.82)	0.66 (0.49–0.82)	–

TKR	Brown hyaena	Black‐backed jackal	Bat‐eared fox	Cape fox
Wet seasons				
Brown hyaena	–	0.56	0.38	0.35
Black‐backed jackal	0.85 (0.74–0.94)	–	0.47	0.47
Bat‐eared fox	0.85 (0.73–0.94)	0.84 (0.74–0.92)	–	0.91
Cape fox	0.86 (0.69–0.98)	0.83 (0.67–0.95)	0.93 (0.80–1.00)	–
Dry seasons				
Brown hyaena	–	0.52	0.39	0.42
Black‐backed jackal	0.80 (0.68–0.90)	–	0.86	0.42
Bat‐eared fox	0.81 (0.68–0.91)	0.88 (0.79–0.95)	–	0.38
Cape fox	0.85 (0.70–0.97)	0.81 (0.67–0.93)	0.81 (0.65–0.94)	–

*Note*: Below the diagonal: Coefficient of overlap, ${\hat{∆}}_{\text{diel}}$ (95% confidence interval). Above the diagonal: Coefficient of overlap in core activity periods, ${\hat{∆}}_{\text{core}}$.
